# Depth-dependent EBIC microscopy of radial-junction Si micropillar arrays

**DOI:** 10.1186/s42649-020-00037-4

**Published:** 2020-09-03

**Authors:** Kaden M. Powell, Heayoung P. Yoon

**Affiliations:** 1 Electrical and Computer Engineering, University of Utah, Salt Lake City, UT 84112 USA; 2 Materials Science and Engineering, University of Utah, Salt Lake City, UT 84112 USA

**Keywords:** EBIC, Electron beam induced current, EBIC efficiency, Radial junction, PN junction, Micropillar array, Solar cells, Local characterization, Scanning electron microscopy

## Abstract

Recent advances in fabrication have enabled radial-junction architectures for cost-effective and high-performance optoelectronic devices. Unlike a planar PN junction, a radial-junction geometry maximizes the optical interaction in the three-dimensional (3D) structures, while effectively extracting the generated carriers via the conformal PN junction. In this paper, we report characterizations of radial PN junctions that consist of *p*-type Si micropillars created by deep reactive-ion etching (DRIE) and an *n*-type layer formed by phosphorus gas diffusion. We use electron-beam induced current (EBIC) microscopy to access the 3D junction profile from the sidewall of the pillars. Our EBIC images reveal uniform PN junctions conformally constructed on the 3D pillar array. Based on Monte-Carlo simulations and EBIC modeling, we estimate local carrier separation/collection efficiency that reflects the quality of the PN junction. We find the EBIC efficiency of the pillar array increases with the incident electron beam energy, consistent with the EBIC behaviors observed in a high-quality planar PN junction. The magnitude of the EBIC efficiency of our pillar array is about 70% at 10 kV, slightly lower than that of the planar device (≈ 81%). We suggest that this reduction could be attributed to the unpassivated pillar surface and the unintended recombination centers in the pillar cores introduced during the DRIE processes. Our results support that the depth-dependent EBIC approach is ideally suitable for evaluating PN junctions formed on micro/nanostructured semiconductors with various geometry.

## Introduction

PN junctions are fundamental device elements that have been extensively used in various applications, including integrated electronic circuits, optical sensors and detectors, and energy harvesting and conversion systems (Chu et al. 2019; Sengupta et al. 1998; Neudeck 1989). Recent advances in micro/nanofabrication have enabled three-dimensional (3D) architectures that offer design flexibility to produce high-performance optoelectronic devices using cost-effective semiconductors (Garnett and Yang 2010; Li 2012; Yoon et al. 2010; Um et al. 2015). These low-quality materials, however, exhibit short minority carrier diffusion lengths (*L*_*n, p*_ < 10 μm) due to high concentrations of impurities and structural defects (e.g., point defect, vacancy, dislocation, grain boundary), limiting device performance designed in a planar geometry. In contrast, a radial-junction configuration maximizes light absorption along the length of the pillars while extracting minority carriers in the radial direction, effectively decoupling the competing processes. The length of the pillars is sufficiently tall to provide adequate light absorption of the indirect bandgap Si absorber, but their diameter allows photo-generated carriers to travel a much shorter distance as compared to traditional structures, increasing the carrier extraction efficiency (Kayes et al. 2005). Additionally, the 3D pillar geometry can be further tailored to reduce reflection and increase light-trapping (Garnett and Yang 2010; Kelzenberg et al. 2010). Our previous work demonstrated over twofold higher power conversion efficiencies with radial junction solar cells compared to their planar junction counterparts (Yoon et al. 2010; Kendrick et al. 2010; Yoon et al. 2011).

Establishing robust junctions is essential for high-performance PN devices, as it controls the flow of excess carriers in one direction, but not the other, providing rectifying characteristics. Various fabrication methods have been proposed and demonstrated to construct the 3D structures with conformal junction formation using top-down (Dowling et al. 2017; Zeniou et al. 2014; Huang et al. 2011; Han et al. 2014; Qian et al. 2020) or bottom-up approaches (Lew and Redwing 2003; Yoo et al. 2013). Plasma-based deep reactive ion etching (DRIE), also known as the “Bosch process” (Laermer and Schilp 2003), has been widely used for 3D patterning owing to the fast and easy processing with reproducible structures. By repeating a cycle of plasma ion etching and conformal polymer coating, DRIE enables the production of high-aspect-ratio structures on various semiconductor substrates. However, the aggressive etching processes often cause unintended surface damage (e.g., porous surface structures, electrically-active defect centers) (Oehrlein 1989; Wu et al. 2010). An inhomogeneous shallow PN junction created on this (sub)surface may introduce poor rectification and inferior diode characteristics. Much of the research activity has been focused on the optimization of fabrication approaches, whereas there are limited studies on the critical PN junction properties of 3D etched Si pillar arrays.

Electron beam induced current (EBIC) microscopy is a powerful analytical technique for studying local electronic states of semiconductor materials and devices (Zhou et al. 2020; Leamy 1982). EBIC uses a focused electron beam to create excess carriers (i.e., electron-hole pairs) near a Schottky or a PN junction. The generated carriers in the quasi-neutral region are diffused in the ambipolar directions. The portion of carriers that reach the junction depends on the recombination rate of their travel path. Subsequently, the local built-in or applied electric field at the junction separates the electron-hole pairs, producing an induced current (i.e., EBIC) in the external circuit. EBIC imaging is frequently used to map excess carrier recombination in semiconductors (Teplin et al. 2015). By fitting an EBIC profile that exponentially decays with distance from the junction, a minority carrier diffusion length can be determined (Yakimov 2015). Moreover, recent studies have proposed advanced EBIC simulations and numerical modeling for quantitative analysis of convoluted EBIC signals (Zhou et al. 2020; Haney et al. 2016).

In this work, we report measurements of radial PN junctions formed on Si micropillar arrays based on depth-dependent EBIC microscopy. By controlling the beam energy that determines the interaction volume between the injected electron beam and the Si structures, we measure the EBIC characteristics of the pillar surface as well as the pillar cores. Our radial PN junctions consist of *p*-type Si micropillar cores fabricated by DRIE and an *n*-type Si shell formed by phosphorous diffusion. We perform EBIC from the sidewall of the pillars to visualize the conformal PN junction. The junction quality of the micropillar array is determined by extracting a local carrier separation/collection efficiency using simulations and EBIC modeling. We compare the EBIC efficiency of the radial junction to the baseline planar PN junction, providing qualitative and quantitive assessment.

## Materials and methods

### Fabrication of Si micropillar arrays

**Fig. 1:**
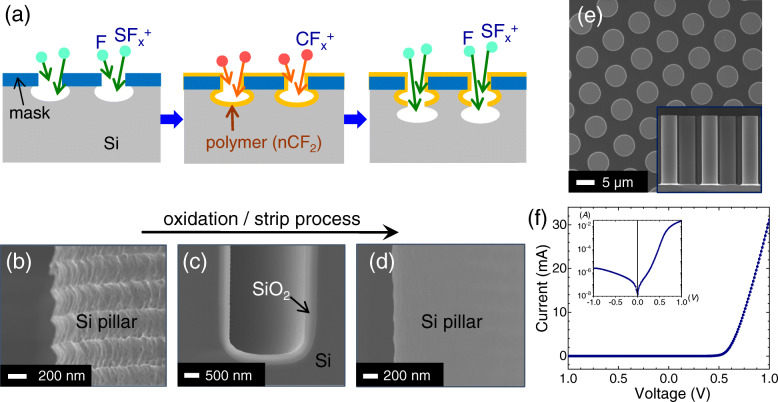
(**a**) Schematics illustrating a single cycle of the deep reactive-ion etching (DRIE) process. (**b**-**d**) SEM images of a pillar sidewall after (**b**) DRIE, (**c**) thermal oxidation, and (**d**) SiO_2_ removal with HF. (**e**) Top-view SEM image of a portion of the complete Si micropillar array. Inset displays cross-section of the device. (**f**) Typical *I-V* curve of pillar array radial junctions, showing ratifying diode behaviors. Inset plots the *I-V* on a log scale

The radial PN junctions studied in this work consist of *p*-type Si micropillar cores fabricated by DRIE and an *n*-type Si shell formed by phosphorous gas diffusion. Details of full fabrication steps can be found elsewhere (Yoon et al. 2010, 2011). Briefly, the process was begun by conventional lithography to pattern a 200 nm thermal oxide layer on Si (*p*-type; *ρ* = 0.01 ~ 0.02 Ω‧cm). The patterned SiO_2_ layer serves as an etch mask in the subsequent anisotropic Si DRIE etching. In DRIE, a cycle of sulfur hexafluoride (SF_6_) etching and octafluorocyclobutane (C_4_F_8_)/oxygen (O_2_) polymer coating (Fig. 1a) was repeated. The C_4_F_8_/ O_2_ polymer coating protects the sidewalls, serving to increase the downward anisotropy of the subsequent SF_6_ etching step and improve verticality of the resulting pillars. Each cycle is composed of (i) 3.5 s SF_6_, (ii) 1.5 s C_4_F_8_ + O_2_, and (iii) 1 s O_2_ at an RF power of 1500 W, providing a Si etch rate of about 2.5 μm/min. To remove the sidewall roughness of the pillar arrays, we used several steps of cleaning processes: (i) O_2_ plasma cleaning (200 sccm O_2_ flow rate, 30 mTorr chamber pressure, and 1800 W RF power for 5 min), (ii) Piranha cleaning (H_2_SO_4_: H_2_O_2_ = 1: 1), and (iii) two successive wet oxidation (1000 °C for 25 min, resulting in ≈ 300 nm thick SiO_2_) and oxide removal processes (10% HF for 1 min). To form radial PN junctions, we used gas phase diffusion of a phosphorus oxychloride (POCl_3_) source at 1000 °C for 13 min. A 300 nm-thick aluminum (Al) metal film was thermally evaporated on the backside of the *p*-type Si and annealed at 600 °C for 10 min in the N_2_ atmosphere. The frontside contacts to the *n*^+^ diffused layers were formed with indium dots on the four corners of the pillar arrays after a native oxide removal in buffered oxide etchant (BOE 1:50, 30 s).

### Si planar PN junction controls

A high-quality Si planar PN device served as a baseline control to evaluate our radial junctions formed on the DRIE-fabricated Si micropillar arrays. We purchased a batch of commercially available Si solar cells (Solar Made) fabricated with high-quality, single-crystalline Si materials. The surface of each device was passivated (e.g., SiN) to reduce the surface recombination velocity. Previously, we conducted a cross-sectional EBIC measurement using this sample. The obtained carrier separation/collection efficiency near the planar PN junction was close to 100%, suggesting a robust PN junction of the planar device (Yoon et al. 2014). In this work, we perform EBIC in a depth-dependent configuration.

### EBIC characterizations

EBIC measurements were carried out in a scanning electron microscope (SEM) equipped with a nano-manipulator. This probe arm was used for the placement of an electrical probe on a metal contact of the device, whereas the bottom metal contact was earthed to the SEM stub. The electrical wires were fitted with coaxial electrical feedthrough of the SEM, connecting to an EBIC current amplifier. Once the electrical contacts were made, a series of EBIC/SEM images were acquired at different incident beam energies using a software package.

### Monte Carlo simulations (e-beam)

We used numerical simulations to estimate the two-dimensional (2D) profile in Si at various incident beam energies (CASINO software package: Monte CArlo SImulation of electroN trajectory in sOlids) (Drouin et al. 2007). In each simulation, we used 200,000 electrons at a fixed accelerating voltage in the range of 1 kV to 30 kV. The injected electrons were propagated in the Si matrix via elastic and inelastic scatterings until their energy becomes 50 eV or less. The radius of the normal incident beam was set to 2 nm. A homogeneous Si substrate in a planar geometry was used for the simulation.

## Results and discussion

Figure 1 displays the key fabrication steps to create Si micropillar arrays. Deep reactive ion etching (DRIE) is a well-established fabrication technique to produce 3D structures by repeating the cycle of a plasma ion etching and a conformal polymer coating (Fig. 1a). These etching/coating processes, however, introduce unintended porous surface structures and electrically-active defect centers. An example of the scalloping profile of the etched structures is shown in Fig. 1b. To remove this structural damage, we used two successive thermal oxidation (1000 °C for 25 min; ≈ 300 nm thick SiO_2_; Fig. 1c) and oxide removal processes (10% HF for 1 min). A representative SEM image in Fig. 1d confirms the dramatically enhanced surface smoothness of the Si micropillars after the rigorous cleaning and oxidation/strip processes. We formed the radial PN junction using gas diffusion of *n*-type phosphorous dopants. An estimated surface doping concentration (*N*_*d*_) is ≈ 10^20^ cm^− 3^ with a junction depth (*x*_*j*_) of ≈ 0.3 μm (Neudeck 1989). The complete Si micropillars measured ≈ 30 μm in height and ≈ 7 μm in diameter with a distance between the pillars of approximately 4 μm (Fig. 1e). The current-voltage (*I-V*) curves of the pillar array in Fig. 1f showed a good diode behavior. We estimated the turn-on voltage of 0.63 V, the leakage current of 10 nA, and the ideality factor of about 1.7. Comprehensive dark and light *I*-*V* characteristics of the radial junctions in various geometrical parameters (e.g., diameter, height, pillar-to-pillar-distance) can be found elsewhere (Yoon et al. 2010, 2011). While extremely informative, *I-V* curves reflect the overall PN junction performance and do not capture the local junction properties.

**Fig. 2:**
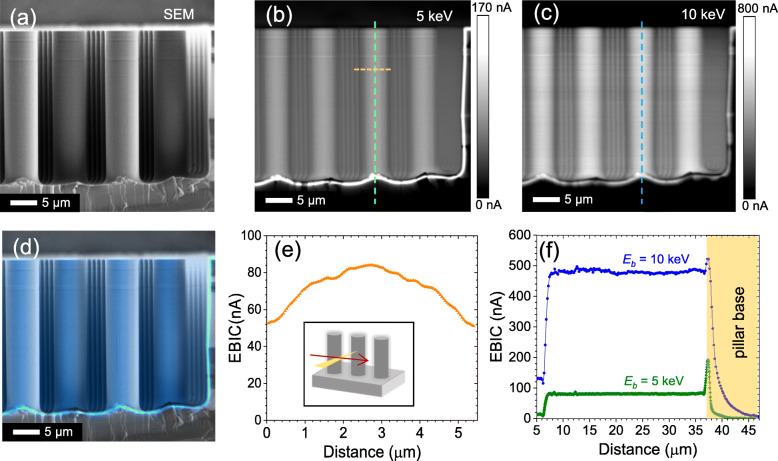
(**a**) SEM image of micropillar array and corresponding EBIC (electron beam induced current) maps at (**b**) 5 keV and (**c**) 10 keV. The relative signal uniformity suggests the presence of conformal radial junctions of individual pillars. (**d**) Overlay of SEM with 5 keV EBIC image confirms continuous PN junction across device. (**e**) Extracted EBIC line scan across pillar diameter. (**f**) EBIC line scans at 5 keV and 10 keV along pillar lengths demonstrate uniform signal magnitude and higher EHP generation at higher energy. Peak EBIC values occur in the pillar base region, where the cross-sectional PN junction is exposed

EBIC microscopy allows a direct access to the local PN junction in 3D with an adjustable probe size from 10’s nm to several μm, in accordance with the interaction volume between the incident electron beam and the semiconductors. To visualize the local junctions at the level of individual pillars, we carefully cleaved the Si pillar array using a fine scriber and exposed the cross-section. The sample was mounted on an EBIC holder, where the metal contacts of the emitter (i.e., indium dots on *n*^*+*^-Si) and the collector (i.e., Al on *p*-Si) were connected to the external EBIC circuit. The electron beam was injected from the *n*-Si emitter shell to the PN depletion region and the *p*-Si pillar core. Figure 2 displays an SEM and the corresponding 5 keV (Fig. 2b) and 10 keV (Fig. 2c) EBIC images. Figure 2d shows the overlaid SEM and 5 keV EBIC, indicating a continuous PN junction formed across the 3D geometry. The overall EBIC intensities of the individual pillars at 5 keV and 10 keV are relatively uniform, suggesting the presence of conformal radial junctions along the individual micropillars.

For a quantitative analysis, we extract the EBIC line scans along/across the pillars. The line scan plot across the pillar diameter (Fig. 2e) shows the highest EBIC value near the pillar center (≈ 80 nA) that decreases gradually with the electron beam probe moving away to the perimeter of the pillar (≈ 50 nA). Considering the direction of the incident electron beam to the curved pillar surface, as illustrated in the inset of Fig. 2e, we suggest that this EBIC change is mainly attributed to the shape of the pillar rather than due to inhomogeneous junction properties. With the increase of the backscattered electrons (BSE) and the decrease of the effective electron-hole pair (EHP) generation volume at the curved pillar surface, the reduction of the EBIC magnitude is evident near the pillar perimeter.

Figure 2f displays the EBIC line scans along the length of the pillars (i.e., axial-direction), showing a relatively constant EBIC value within the pillars at a fixed accelerating voltage. The mean EBIC value of the pillar increases from 80 nA at 5 keV to 480 nA at 10 keV. We observe the highest EBIC values are present in the area of the pillar base, which is associated with the direct local carrier generation within the depletion region. When the electron beam is directly injected to the cross-sectional PN junction (i.e., mechanically cleaved junction area), the generated EHPs are separated quickly without diffusion owing to the built-in electric field (C. J. Wu and Wittry 1978; Yakimov 2015). In contrast, the electron beam irradiated on the pillar sidewalls generates the EHPs in the depletion region as well as the charge-neutral regions (i.e., *n*-Si shell, *p*-Si pillar core). The excess carriers must travel to the junction (i.e., ambipolar diffusion) before they are separated and collected in EBIC. Since the *n*-Si emitter layer (*N*_*d*_ ≈ 10^20^ cm^− 3^, *x*_*j*_ ≈ 300 nm) of our pillars is conductive, yet highly defective, the EHPs generated in this region tend to be recombined, decreasing overall EBIC values as compared to the direct electron beam injection at the cross-sectional PN junction. As the EHP generation volume increases with the accelerating voltage (i.e.*,* 5 keV to 10 keV), the portion of the EHPs generated in the *n*-Si decreases, resulting in a comparable EBIC value of the pillar and near the base.

**Fig. 3:**
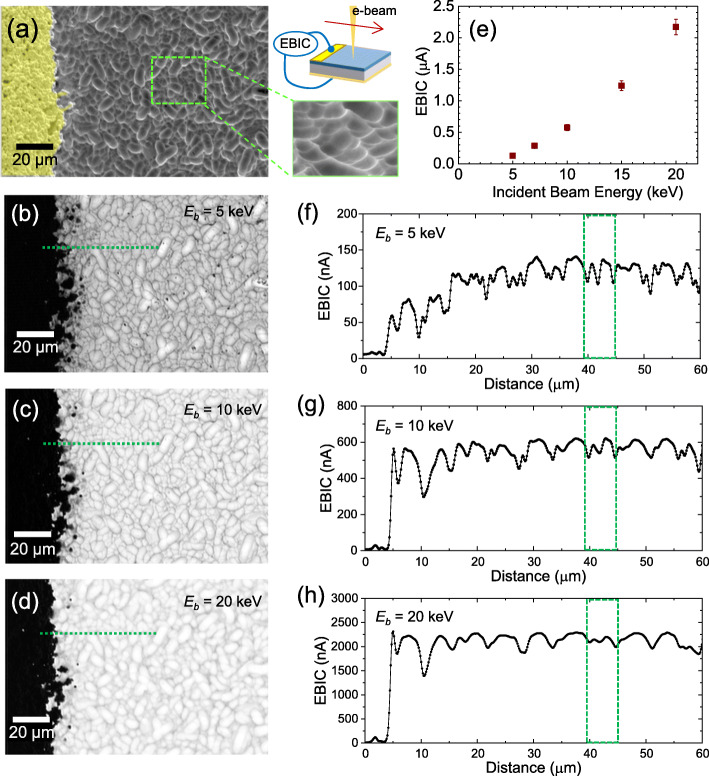
(**a**) SEM image of planar PN device with metal contact highlighted in yellow. [center top] Schematic of EBIC measurement. [center bottom] Slightly tilted SEM image of the sample, illustrating surface roughness. Corresponding EBIC scans of the planar device at (**b**) 5 keV, (**c**) 10 keV, and (**d**) 20 keV showing higher contrast and reduced spatial resolution with higher energy. (**e**) Increasing mean EBIC current calculated for Si line scans. (**f**-**h**) Plots of EBIC current along line scans shown with green lines in (**b**-**d**): (**f**) 5 keV, (**g**) 10 keV, (**h**) 20 keV. The green box highlights the representative sample topography. The two distinct features at 5 keV become broad and indistinguishable with higher beam energies

To assess the local junction quality of the micropillar array, we collected the baseline EBIC characteristics of a commercial planar device (Solar Made). This planar PN junction (*n*^+^-*p*) was built on a high-purity single crystalline Si substrate, and it showed a carrier collection efficiency close to 100% in the depletion region obtained in a normal collector EBIC configuration (Yoon et al. 2014). Figure 3 displays a representative SEM image of the planar PN device and the corresponding EBIC maps collected at 5 keV, 10 keV, and 20 keV. The large dark area of the EBIC images is associated with the metal contact, highlighted in yellow in the SEM image (Fig. 3a). The injected electron beam (1 keV ~ 30 keV) does not penetrate this thick metal layer (a few mm thick Ag paste), producing negligible EBIC signals (Fig. 3b-d). The dark speckles in the 5 keV EBIC image are likely attributed to thin organic residue or dust particles on the sample, of which EBIC contribution becomes insignificant with the higher beam energies (> 10 keV). Qualitatively, the bright contrast increases with a higher keV, showing similar behaviors as those observed with the pillar array radial junction (Fig. 2).

Figure 3f through g show the representative line scans extracted from the EBIC images (Fig. 3b-d). A relatively constant EBIC was observed in the device area, a stack of *p*-Si collector, *n*-Si emitter, and SiN passivation layer. A notable current fluctuation near the metal contact is mainly attributed to the spread of the metal paste. By aligning the line scans, we find that a low keV EBIC is much more sensitive to the surface features than higher keV. For instance, two distinct peaks observed in the 5 keV EBIC line scan (marked with a green box) conform to the sample topography shown in SEM (Fig. 3a). This feature becomes less distinguishable with increasing incident beam energy, as the electron beam penetrates deep in the sample with a larger EHP generation volume. We used the EBIC images from 5 keV to 30 keV and calculated mean EBIC values for the Si area, shown in Fig. 3e. The increase of EBIC with a higher keV is evident in the line scans. The average EBIC value increases from 127 nA at 5 kV to 3.55 μA at 30 kV, increasing over one order of magnitude. Interestingly, the EBIC values observed in the planar junction are slightly higher than those in the radial junction in Fig. 2: 127 nA (vs. 83 nA of the radial junction) at 5 keV, 574 nA (vs. 492 nA of the radial junction) at 10 keV.

**Fig. 4:**
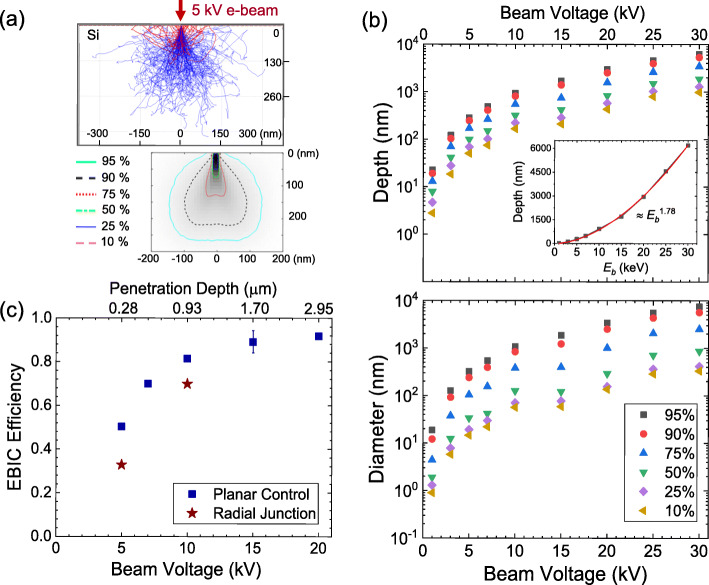
(**a**) Simulated electron paths for a ray of 5 kV electron beam in Si (200,000 electrons). Blue lines represent collision events of electrons with Si. Red lines represent backscattered electrons. (**a**, bottom) Energy contour plot from 5 kV simulation with percent energy loss contours. (**b**) Simulated interaction bulb size at various accelerating voltages: [top] maximum depth and [bottom] maximum diameter. Inset shows the penetration depth with beam energy (95% energy contour) and corresponding curve fit. (**c**) Estimated EBIC efficiency of the radial junction compared to the planar PN junction control

The experimental results qualitatively suggest that EBIC magnitude near the PN junctions is strongly influenced by the EHP generation by the incident electron beam and the local carrier separation/collection properties. To gain a deeper understanding of the local radial junction characteristics, we estimate the carrier generation profile using Monte Carlo simulations and calculate the local carrier collection efficiency for the planar and the radial junctions. Figure 4a (top) displays an example of the simulated electron trajectories, where a ray of 5 keV electron beam is irradiated onto a Si substrate. The blue lines represent the collision events of the primary electrons with Si until they lose their initial energy (i.e., 5000 V in this example) to 50 V or lower. The red lines represent the paths of the backscattered electrons. A corresponding energy contour plot is shown in the bottom image. For instance, the 95% contour represents the sample area where the injected primary electrons have lost 95% of their initial energy. Figure 4b plots the estimated interaction bulb size at different accelerating voltages (1 kV ~ 30 kV). The overall ratio of depth to diameter (depth/diameter) is comparable for higher lost-energy contours (> 75%), yet slightly higher for low energy contours (< 50%), indicating a pear-shape of the interaction bulb. The calculated bulb size at 1 keV is approximately 19 nm, inferring that the spatial resolution of the EBIC image for flat Si devices can be achieved as high as < 20 nm. The inset of Fig. 4b shows the increase of the penetration depth with the beam energy, which was extracted from the 95% energy contour of each simulation. The numerical fit overlaid on the datasets confirms the bulb size is proportional to ${E}_b^{1.78}$ (*E*_*b*_ is the beam energy), showing an excellent agreement with the analytical prediction of $\approx {E}_b^{1.7}$ by Wittry et al. (Wittry and Kyser 1967). By controlling the incident beam energy, the size of the EHP generation bulb can be tunable from 10’s nm to several μm, offering versatility to study local carrier dynamics in optoelectronic semiconductor materials and devices.

Based on the Monte-Carlo simulations and EBIC modeling (Leamy 1982; Haney et al. 2016; Yakimov 2015), we estimated the local carrier separation/carrier efficiency of the radial junction and compared it to planar PN controls. The EBIC collection efficiency (*η*_*EBIC*_) is defined as the ratio of the measured current (*I*_*EBIC*_) to the EHP generation rate (*β*). Here, *e* is the unit charge (1.6 × 10^− 19^ C).

1 $$ \eta (EBIC)=\frac{I_{EBIC}}{e\cdot \beta}\kern0.5em $$

The generation rate, which is the total number of EHPs created by the injected electron beam, can be calculated as below.

2 $$ \beta =\frac{I_b\cdot {E}_b\cdot \alpha }{e\cdot {E}_{EHP}} $$

*E*_*b*_ is the incident electron beam energy, *α* is the fraction of beam energy absorbed inside the material (i.e., Si in our case), and *E*_*EHP*_ is the average energy to create an electron-hole pair. The *I*_*b*_ of our SEM was measured in the range of 250 pA (*E*_*b*_ = 5 kV) to 300 pA (*E*_*b*_ = 20 kV). We calculated the magnitude of *α* using the backscattered coefficient obtained from the Monte Carlo simulation (e.g., 0.152 at 5 keV, 0.142 at 20 keV). The *E*_*EHP*_ was estimated using an empirical relation of *E*_*EHP*_ = 2.596 *E*_*g*_ + 0.174 (Kobayashi et al. 1972), giving *E*_*EHP*_ ≈ 3.621 *eV* for Si (*E*_*g*_ = 1.12 *eV*). The EBIC currents (*I*_*b*_) extracted from the line scans in Figs. 2 and 3 were used for the pillar array and the planar device, respectively. We note that a typical uncertainty in our EBIC measurement and analysis is about 10% associated with the fluctuations of the baseline e-beam current (*I*_*b*_) and the signal-to-noise ratio of the EBIC preamplifier. Also, the parameters extracted from the Monte Carlo simulations (e.g., backscattered coefficient, mean EHP generation rate (*β*), empirical parameter (*E*_*EHP*_) to generate EHPs) contribute to the uncertainty.

Finally, we plot the resulting EBIC efficiency of the devices at different incident beam energies in Fig. 4c. The EBIC efficiency increases with the incident beam energy, reaching close to unity at *E*_*b*_ > 15 keV for the planar PN junction device. A similar trend was observed for the radial junction of the pillar array, yet the overall EBIC efficiency is slightly lower than that of the planar device (about 10%). In both cases, EBIC was measured in the depth-dependent configuration. The injected electrons travel from the highly-doped emitter (a few 100 nm thick) to the depletion region (< 1 μm) and the *p*-Si collector, generating the EHPs in three different layers. The low EBIC efficiency at 5 keV (50% for the planar device; 30% for the pillar array) is likely attributed to the EHP production in the highly-doped emitter region. Our Monte Carlo simulation shows an interaction bulb size of (300 nm)^3^ at 5 keV, suggesting that most EHPs were produced in the highly-defective (i.e., high-density of recombination centers) emitter region that promotes excess carrier recombination. At higher keV, most EHPs are generated in the strong built-in electric field region and the collector, increasing the EBIC efficiency. Our observation indicates that the surface damage introduced on the pillars by DRIE could be effectively removed by rigorous cleaning and oxidation/strip processes. The magnitude of the EBIC efficiency of our pillar array is about 70% at 10 kV, slightly lower than that of the planar device (≈ 81%). We speculate that a slightly higher EBIC efficiency for the planar junction is associated with the surface passivation (e.g., SiN) that decreases the surface recombination of EHPs. Detailed EBIC studies of the surface passivation based on 3D continuity equations together with Poisson equations (Yakimov 2015; Haney et al. 2016; Zhou et al. 2020) will provide additional insight on the excess carrier dynamics and general guidance to improve their device performance.

## Summary and conclusions

In summary, we have examined the radial junction characteristics of Si micropillar arrays using depth-dependent EBIC microscopy. The EBIC images collected from the sidewall of the pillars confirm the uniform PN junction conformally constructed on the 3D pillar array. We find the EBIC efficiency of the pillar array increases with the injected electron-beam voltage, consistent with the EBIC behaviors observed in a high-quality planar PN junction. The magnitude of the EBIC efficiency of our pillar array is about 70% at 10 kV, slightly lower than that of the planar device (≈ 81%). We suggest that this reduction could be attributed to the unpassivated pillar surface or the low material quality of the pillar core. Our results support that the depth-dependent EBIC approach is ideally suitable for evaluating 3D conformal PN junctions formed on micro/nanostructures with various geometries.

## Data Availability

The datasets used in this study are available from the corresponding author on reasonable request.

## Abbreviations

2D: Two-dimensional
3D: Three-dimensional
BSE: Backscattered electron
CASINO: Monte-CArlo SImulation of electroN trajectory in sOlids
DRIE: Deep reactive-ion etching
EBIC: Electron-beam induced current
EHP: Electron-hole pair
RF: Radio frequency
SEM: Scanning electron microscopy
